# Human iPSCs Derived MSCs‐Secreted Exosomes Modulate Senescent Nucleus Pulposus Cells Induced Macrophage Polarization via Metabolic Reprogramming to Mitigate Intervertebral Disc Degeneration

**DOI:** 10.1002/advs.202504347

**Published:** 2025-07-06

**Authors:** Qian Xiang, Jiawen Zhan, Shuo Tian, Yongzhao Zhao, Zhenquan Wu, Jialiang Lin, Longting Chen, Longjie Wang, Shuai Jiang, Zhuoran Sun, Weishi Li

**Affiliations:** ^1^ Department of Orthopaedics Peking University Third Hospital Beijing 100191 China; ^2^ Engineering Research Center of Bone and Joint Precision Medicine Ministry of Education Beijing 100191 China; ^3^ Beijing Key Laboratory of Spinal Disease Research Beijing 100191 China; ^4^ Department of Orthopaedics Wangjing Hospital China Academy of Chinese Medical Sciences Beijing 100102 China

**Keywords:** intervertebral disc degeneration, induced pluripotent stem cells, exosomes, macrophage polarization, metabolic reprogramming

## Abstract

Intervertebral disc degeneration (IDD) is a leading cause of discogenic lower back pain, yet the crosstalk between macrophage polarization and nucleus pulposus (NP) cell senescence in IDD progression remains poorly understood. Emerging therapies using human induced pluripotent stem cell (iPSCs)‐derived mesenchymal stem cells (iMSCs) show promise for IDD treatment. In this study, it is first demonstrated that senescent NP cells promote macrophage polarization toward the pro‐inflammatory M1 phenotype in coculture systems. Reciprocally, conditioned medium from M1 macrophages exposed to senescent NP cells accelerates senescence in healthy NP cells. Notably, it is identified that iMSCs‐derived exosomes break this pathogenic cycle by reprogramming M1 macrophages toward anti‐inflammatory M2 phenotypes. Mechanistically, these exosomes deliver miR‐100‐5p to suppress mTORC1 signaling and regulate glycolysis metabolic reprogramming in macrophages. These findings are corroborated in a rat IDD model, where iMSC‐exosomes mitigate IDD progression in vivo. This work elucidates a novel iMSC‐exosomes mediated mechanism regulating macrophage‐NP cell interactions, which provides a promising therapeutic strategy for IDD intervention.

## Graphical abstract

Senescent nucleus pulposus cells drive inflammatory M1 macrophage polarization, exacerbating intervertebral disc degeneration (IDD). Conversely, M1 macrophages accelerate NP cell senescence, creating a vicious cycle. iPSCs‐derived mesenchymal stem cell (iMSCs) exosomes break this loop by delivering miR‐100‐5p to suppress mTORC1/glycolysis in macrophages, thus mitigating IDD progression—revealing a novel cell‐free therapeutic strategy.

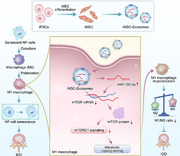

## 1 Introduction

Intervertebral disc degeneration (IDD) represents a prevalent degenerative spinal disorder and a primary etiology of discogenic low back pain, often leading to chronic pain, functional impairment, and even disability.^[^ ^1^, ^2^ ^]^ The intervertebral disc (IVD) consists of three distinct anatomical regions: the central nucleus pulposus (NP), surrounding annulus fibrosus (AF), and cartilaginous endplates (EP), and IVD serves essential biomechanical functions in load distribution and spinal mobility. Current evidence suggests that NP degeneration constitutes the initial pathological trigger in IDD progression.^[^ ^3^ ^]^ Pathologically, the NP is replaced by fibroblast‐like phenotype cells with an imbalance of catabolism and anabolism of the disc extracellular matrix in the degeneration progression.^[^ ^4^, ^5^ ^]^ Besides, the IDD process is accompanied by annulus fibrosus disorder or rupture, leading to immune cell infiltration and inflammation response.^[^ ^6^ ^]^ Critically, IDD pathogenesis is intrinsically linked to the biological fate of NP cells.^[^ ^7^ ^]^ Particularly, NP cell senescence leads to the disruption of the IVD homeostasis, thereby serving as a pivotal driver of IDD progression.^[^ ^8^ ^]^ In the process of cell senescence, the cell proliferation capacity is decreased, accompanied by elevated levels of SA‐β‐gal activity, and the induction of cellular senescence‐related markers, including p16 and p21.^[^ ^9^, ^10^ ^]^

Macrophages are widely distributed in the human body as the main phagocytic immune cells, and they could infiltrate into the structurally injured areas of the IVD with degeneration.^[^ ^11^ ^]^ Accumulating evidence has revealed that macrophages‐based inflammatory micro‐environment in degenerated IVD significantly promotes NP cell degeneration and deterioration.^[^ ^12^, ^13^ ^]^ Macrophages could secrete various inflammatory mediators, including TNF‐α, IL‐1β, and IL‐6, which promoted the disc cell phenotype changes, and thus to result in IDD progression.^[^ ^14^, ^15^ ^]^ The activated macrophages can be divided into two main populations: pro‐inflammatory M1 cells and anti‐inflammatory M2 cells. The macrophage polarization to M1 or M2 phenotypes is closely associated with metabolism changes (metabolic reprogramming), and M1‐type macrophages primarily rely on glycolysis. In contrast, M2‐polarized macrophages primarily rely on oxidative phosphorylation (OXPHOS) and fatty acid oxidation (FAO) for energy metabolism.^[^ ^16^ ^]^ Metabolic reprogramming from glycolysis toward FAO has been found to suppress M1 macrophages activation while enhancing M2 macrophage polarization, highlighting the critical role of energy metabolism in determining macrophages functional states.^[^ ^17^ ^]^ Recent research has validated that M1 phenotype macrophages infiltration in degenerated disc NP tissues was markedly up‐regulated.^[^ ^18^ ^]^ In this study, we investigated the interaction between macrophage polarization and the nucleus pulposus cell senescence for the first time. Interestingly, we found that senescent nucleus pulposus cells significantly promoted macrophages polarizing into the pro‐inflammatory M1 phenotype. Furthermore, senescent NP cells‐induced M1 macrophages derived conditioned medium could promote NP cell senescence in return.

Exosomes are a kind of natural extracellular vesicles with high biocompatibility and low toxicity, which play a key role in intercellular communication.^[^ ^19^, ^20^ ^]^ Mesenchymal stem cell (MSCs) ‐derived exosomes possess many regenerative bioactive molecules and have exhibited therapeutic potentials in degenerative musculoskeletal diseases including disc degeneration diseases.^[^ ^21^, ^22^ ^]^ However, the clinical application of MSCs faces several limitations, including invasive harvesting procedures, limited consistency, and restricted proliferative capacity. Interestingly, iMSCs are a novel type of MSCs derived from induced pluripotent stem cells (iPSCs), which offer a superior alternative due to their enhanced proliferation capacity, standardized production, and reduced donor variability.^[^ ^23^ ^]^ Moreover, iMSCs bypass ethical concerns and immune rejection risks while exhibiting robust expansion potential, enabling large‐scale production of therapeutic exosomes. Given their regenerative properties and scalable production, iMSC‐derived exosomes emerge as a viable cell‐free therapy for intervertebral disc degeneration, with imminent clinical prospects.

Considering the critical roles of macrophages in IDD and the therapeutic potentials of iMSCs derived exosomes (iMSCs‐Exos) in disc degeneration diseases, we aimed to systematically investigated the function and mechanisms of iMSCs‐Exos on macrophage polarization and NP cell senescence in this study. We found that iMSCs‐Exos could promote the transition of senescent NP cells‐induced M1 macrophages into anti‐inflammatory M2 phenotype. In mechanisms, iMSCs‐Exos transported the most abundantly enriched miRNA miR‐100‐5p, which targeted the mammalian target of rapamycin (mTOR) and inhibited the mTORC1 signaling to regulate glycolysis in macrophages. These findings were further validated in vivo by using a rat IDD model. Our study shed light on the critical roles of iMSCs‐Exos in modulating the macrophage polarization and glycolysis metabolic reprogramming, which might provide a promising therapeutic strategy for IDD treatment.

## 2 Results

### 2.1 M1‐Type Macrophages Proportion and Cell Senescence were Elevated in IDD

**Figure 1:**
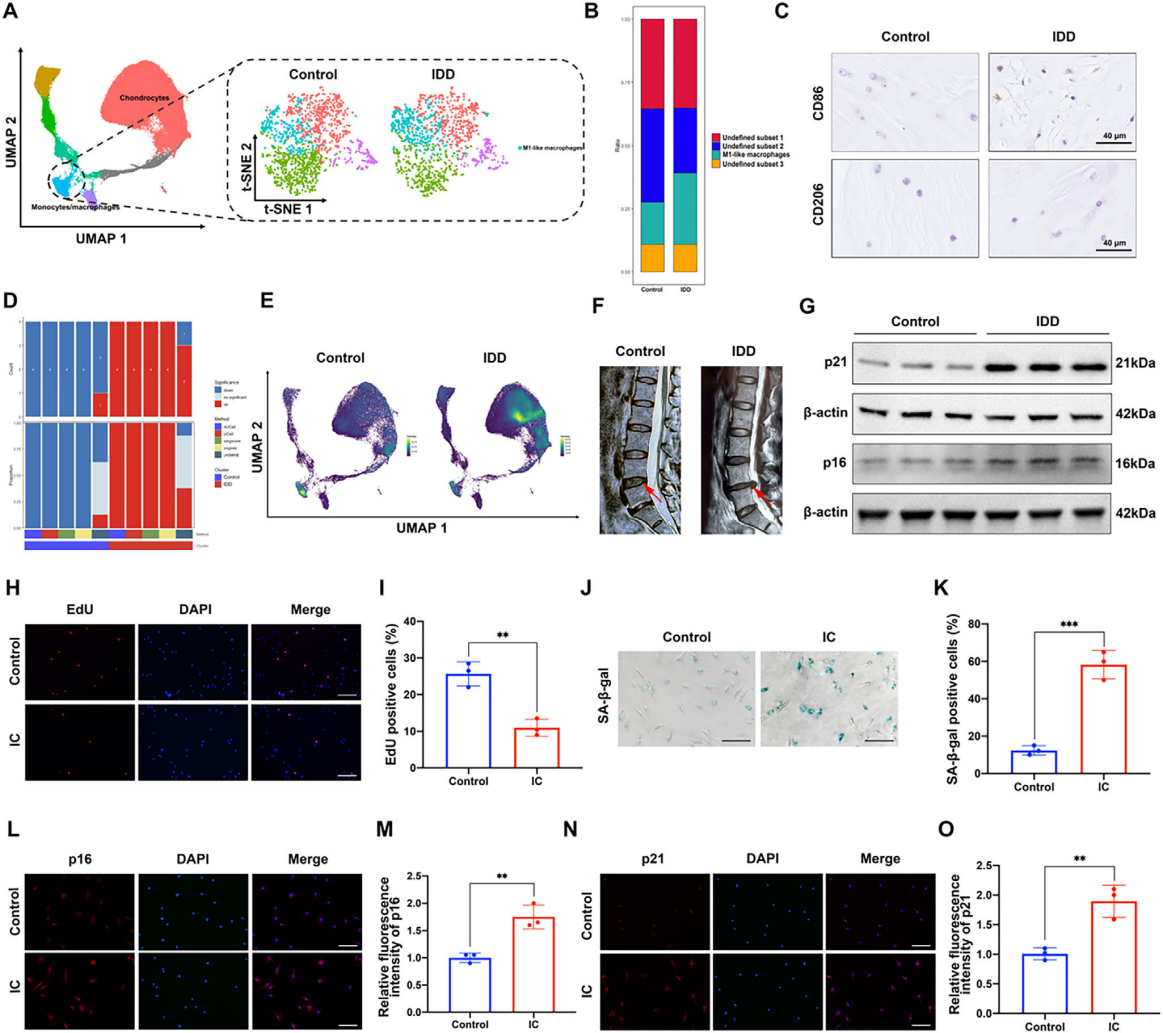
The cell senescence score and M1 macrophages proportion are up‐regulated in IDD. A) T‐SNE analysis plot of single‐cell transcriptome datasets (GEO: GSE205535, GSE153066; CNGBdb: CNP0002664) identified by SingleR. B) Monocytes/macrophages subpopulations are annotated by Sctype based on annotated marker genes. C) Immunohistochemical staining of macrophages M1 phenotype marker CD86, and M2 phenotype marker CD206 in control and degenerated human NP tissues. D) Relative cell senescence levels in control and degeneration groups assessed by different methods including AUCell, UCell, singscore, ssgsea, JASMINE. E) Heat map based on the density of AUCell enriched SenMayo senescence scores. F) The MRI T2‐weighted images of control and degenerated IVDs. G) The protein expression level of p21 in human NP tissues of control and degeneration groups was assessed by western blot analysis. H,I) The cell proliferation of control and senescent NP cells was evaluated by EdU staining analysis, with the EdU‐positive cells quantified. Scale bar: 100 µm. J,K) SA‐β‐gal staining analysis was performed in the human NP cells of the corresponding group, with the SA‐β‐gal positive cells quantified. Scale bar: 50 µm. L,M) The protein expression level of p16 in human NP cells of each group was determined by immunofluorescence staining, with the relative intensity of fluorescence quantified. Scale bar: 50 µm. N,O) The protein expression level of p21 in human NP cells of each group was determined by immunofluorescence staining, with the relative intensity of fluorescence quantified. Scale bar: 50 µm. Data were shown as means with error bars representing SD. ^**^ *p* < 0.01, ^***^ *p* < 0.001, n = 3.

To investigate the association between IDD and macrophage polarization, we analyzed single‐cell RNA sequencing datasets (GEO: GSE205535, GSE153066; CNGBdb: CNP0002664) by using SingleR. We found the M1‐like phenotype macrophages proportions were different in the NP regions of the control and IDD group (**Figure** **1**A). The proportion of M1‐like macrophages was significantly elevated in the IDD group compared to control (Figure 1B). Moreover, immunohistochemical staining analysis further confirmed that the proportion of M1 phenotype macrophages was significantly elevated in the degenerated human NP tissues compared to control, while the proportion of M2 phenotype macrophages was very low in both groups (Figure 1C; Figure S1A, Supporting Information). Then we analyzed the cell senescence score in IDD and control group. Results showed that the relative cell senescence level was obviously increased in the degeneration group assessed by different ordinary methods (Figure 1D). The Heat map based on the density of AUCell enriched SenMayo senescence score evidently showed the elevated cell senescence level in IDD (Figure 1E). Next, immunoblotting analysis of human NP tissues revealed that the senescence marker p21 protein expression level was markedly up‐regulated in degenerated disc tissues compared with control (Figure 1F,G; Figure S1B, Supporting Information). Subsequently, inflammatory cytokines (20 ng mL^−1^ of TNF‐α and IL‐1β) administration was applied to establish a senescent NP cells model in vitro. The inflammatory cytokines (IC) treatment significantly induced NP cell senescence, based on the decreased cell proliferation level (Figure 1H,I), and increased SA‐β‐gal activity in IC group (Figure 1J,K). The flow cytometry analysis was performed to evaluate the cell cycle. Compared to the control group, IC treatment significantly increased the cell proportion of G0/G1 phase, and decreased the cell proportion of the S phase (Figure S2, Supporting Information). Besides, the immunofluorescence results also validated that the expression levels of senescence markers p16 and p21 were significantly up‐regulated in IC induced senescence group (Figure 1L–O). These data indicated that IC treatment significantly induced NP cells senescence in vitro.

### 2.2 Senescent NP Cells‐Induced M1 Macrophages Derived Conditioned Medium Could Promote NP Cell Senescence

**Figure 2:**
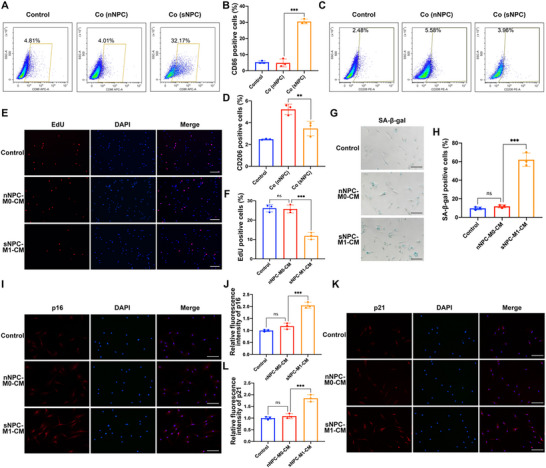
Senescent NPC coculture‐induced M1 macrophages conditioned medium promoted NP cells senescence. A,B) The flow cytometry analysis and quantification of CD68^+^ macrophages in control group, normal NPC coculture group, and senescent NPC coculture group. C,D) The flow cytometry analysis and quantification of CD206^+^ macrophages in control group, normal NPC coculture group, and senescent NPC coculture group. E,F) The cell proliferation of NP cells treated with conditioned medium of normal NPC coculture‐induced M0 macrophages, or senescent NPC coculture‐induced M1 macrophages, was evaluated by EdU staining analysis, with the EdU positive cells quantified. Scale bar: 100 µm. G,H) SA‐β‐gal staining analysis was performed in the human NP cells of each group, with the SA‐β‐gal positive cells quantified. Scale bar: 50 µm. I,J) The protein expression level of p16 in human NP cells of each group was detected by immunofluorescence staining, with the relative intensity of fluorescence quantified. Scale bar: 50 µm. K,L) The protein expression level of p21 in human NP cells of each group was detected by immunofluorescence staining, with the relative intensity of fluorescence quantified. Scale bar: 50 µm. Data were shown as means with error bars representing SD. ns, not significant; ^**^ *p* < 0.01, ^***^ *p* < 0.001, n = 3.

To explore the interaction between macrophages and the nucleus pulposus cell, we first investigated the effects of senescent NP cells on the polarization of macrophages. The M0 macrophages were cocultured with normal NP cells (nNPC) or senescent NP cells (sNPC) for 24 h, and then were collected for macrophage polarization analysis by flow cytometry analysis. As shown in **Figure** **2**A,B, the CD86 positive cells proportion was significantly increased in sNPC coculture group compared to nNPC coculture group, suggesting that senescent NP cells could induce macrophages M1 polarization. However, the CD206 positive cell proportion was slightly decreased in sNPC coculture group compared to nNPC coculture group, indicating senescent NP cells could also inhibit macrophages M2 polarization (Figure 2C,D). Then we further investigated the roles of M1 macrophages conditioned medium (CM) on NP cells. As revealed by the EdU analysis, the sNPC‐treated macrophages‐CM markedly suppressed the NP cell proliferation level compared to the nNPC‐treated macrophages‐CM (Figure 2E,F). The SA‐β‐gal activity was also elevated in sNPC‐M1‐CM group (Figure 2G,H). The cell cycle analysis revealed that compared to nNPC‐treated macrophages‐CM group, sNPC‐treated macrophages‐CM markedly increased the cell proportion of G0/G1 phase, and decreased the cell proportion of the S phase (Figure S3, Supporting Information). Besides, the immunofluorescence analysis of p16 and p21 suggested that sNPC‐M1‐CM markedly promoted the NP cell senescence (Figure 2I–L). Interestingly, the above results revealed that senescent NP cells‐induced M1 macrophages conditioned medium could promote NP cell senescence in return. In other words, NP cells senescence could be transmitted and expanded by macrophage polarization.

### 2.3 iMSC‐Exos Mitigated IDD Progression and NP Cells Senescence In Vivo

**Figure 3:**
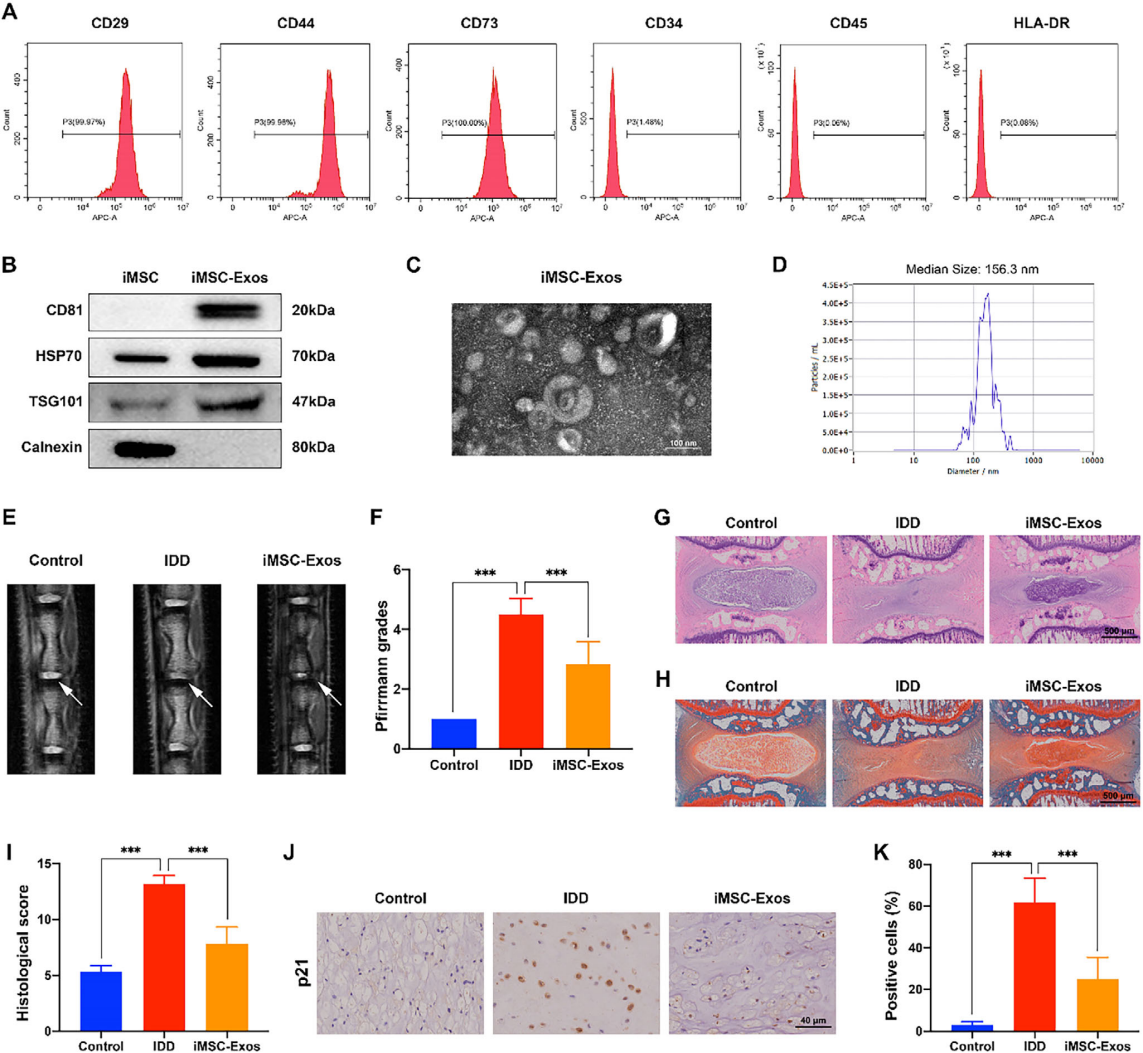
Intradiscal injection of iMSC‐Exos mitigated IDD progression and NP cell senescence in vivo. A) The surface phenotype markers of iMSC were evaluated by flow cytometry analysis, with positive expression of CD29, CD44, CD73, and negative expression of CD34, CD45, and HLA‐DR. B) The surface markers of iMSC‐Exos were examined by western blot analysis, with positive expression of CD81, HSP70, and negative expression of Calnexin. C) The morphology of iMSC‐Exos was detected by TEM. Scale bar: 100 nm. D) The diameter and concentration of iMSC‐Exos were detected by NTA analysis. E) The IVD from the rat tail from each group was evaluated by MRI at T2‐weighted signal (white arrows). F) Quantitation of the degree of IDD based on the Pfirrmann grade system using MRI results. G) HE staining of the rat tail IVD from each treatment group. Scale bar: 500 µm. H) SO staining of the rat tail IVD from each treatment group. Scale bar: 500 µm. I) The histological score of the rat tail IVD based on the histological grading scale. J,K) The protein expression level of p21 in the rat tail IVD from each treatment group was determined by immunohistochemical staining. Scale bar: 40 µm. ^***^ *p* < 0.001, n = 6.

The iPSC‐derived mesenchymal stem cells (iMSCs) secreted exosomes have emerged as a promising strategy for degenerative musculoskeletal diseases and other human diseases. First, the flow cytometry analysis validated that iMSCs surface markers CD29, CD44, and CD73 were positive, while CD34, CD45, and HLA‐DR were negative (**Figure** **3**A). Then the iMSCs‐derived exosomes were extracted and characterized. Western blotting results revealed that the iMSC‐Exos expressed exosomes markers CD81 and HSP70, and did not express the negative marker Calnexin (Figure 3B). Transmission electron microscopy (TEM) results showed that iMSC‐Exos had a typical cup‐like appearance (Figure 3C). Nanoparticle tracking analysis (NTA) indicated the sizes of iMSC‐Exos were ≈100 to 200 nm (Figure 3D). By using an IDD rat model, we then investigated the effects of iMSC‐Exos on IDD progression in vivo. The IVD degeneration degree was evaluated by MRI and Pfirrmann grading scores.^[^ ^24^ ^]^ The MRI results suggested that the T2‐weighted signal value was higher in iMSC‐Exos treated group compared to IDD group (Figure 3E), and the Pfirrmann MRI grading score was lower in iMSC‐Exos treated group compared to IDD group (Figure 3F). Then HE and SO staining analysis were applied to assess the histomorphological changes in more detail. The disc in the IDD group showed a collapse of the disc height and fibrous‐tissue invasion compare to the control disc. However, iMSC‐Exos injection significantly mitigated the NP tissue collapse and the disc structure destruction (Figure 3G,H). The histological score also revealed the protecting effects of iMSC‐Exos on the disc (Figure 3I). Subsequent immunohistochemical analysis of cell senescence marker p21 in the disc NP demonstrated that iMSC‐Exos could inhibit cell senescence in IDD (Figure 3J,K). Collectively, the above results showed that iMSC‐Exos alleviated IDD progression in vivo.

### 2.4 iMSCs‐Exos Regulated the Re‐Polarization of sNPC‐Induced M1 Macrophages to Suppress NP Cells Senescence

**Figure 4:**
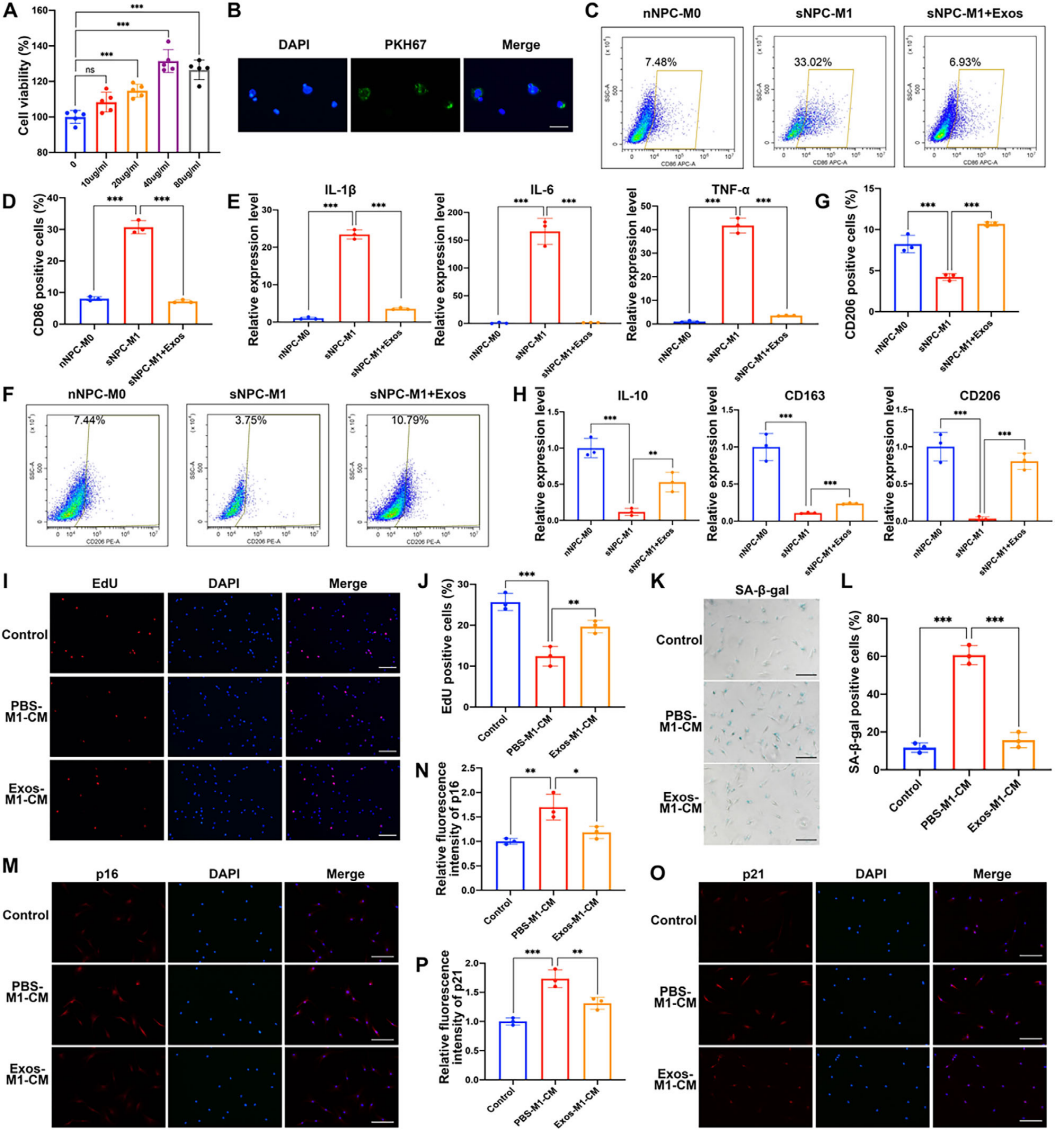
The iMSCs‐Exos regulated the re‐polarization of senescent NPC coculture‐induced M1 macrophages into anti‐inflammatory M2 macrophages. A) The cell viability of macrophages treated with different concentration of iMSCs‐Exos was analyzed by CCK‐8 assay. ns, not significant; ^***^ *p* < 0.001, n = 5. B) Representative immunofluorescence image of the internalization of PKH67‐labeled iMSCs‐Exos in macrophages. Scale bar: 20 µm. C,D) The flow cytometry analysis and quantification of CD68^+^ macrophages in control, senescent NPC coculture‐induced M1 macrophages without or with iMSCs‐Exos treatment groups. E) The mRNA expression level of IL‐1β, IL‐6, and TNF‐α of the macrophages from each group was determined by RT‐qPCR analysis. F,G) The flow cytometry analysis and quantification of CD206^+^ macrophages in each treatment group. H) The mRNA expression level of IL‐10, CD163, and CD206 of the macrophages from each group was determined by RT‐qPCR analysis. I,J) The NP cells were treated with conditioned medium of M0 macrophages, M1 macrophages, or iMSCs‐Exos pre‐treated M1 macrophages, and the cell proliferation of NP cells of each group was evaluated by EdU staining analysis, with the EdU positive cells quantified. Scale bar: 100 µm. K,L) SA‐β‐gal staining analysis was performed in the human NP cells of each group, with the SA‐β‐gal positive cells quantified. Scale bar: 50 µm. M,N) The protein expression level of p16 in human NP cells of each group was detected by immunofluorescence staining, with the relative intensity of fluorescence quantified. Scale bar: 50 µm. O,P) The protein expression level of p21 in human NP cells of each group was detected by immunofluorescence staining, with the relative intensity of fluorescence quantified. Scale bar: 50 µm. Data were shown as means with error bars representing SD. ^*^ *p* < 0.05, ^**^ *p* < 0.01, ^***^ *p* < 0.001, n = 3.

Then we further investigated the effects of iMSCs‐Exos on polarization of macrophages induced by senescent NP cells. The CCK‐8 analysis revealed that iMSCs‐Exos treatment could increase the proliferation of macrophages to an extent (**Figure** **4**A). The PKH67‐labeled iMSCs‐Exos were confirmed to be endocytosed into the macrophages after incubation for 12 h (Figure 4B). A subsequent series of experiments was performed to detect the effects of iMSCs‐Exos on macrophage polarization. The flow cytometry analysis of M1 polarization marker CD86 results showed that iMSCs‐Exos significantly inhibited the macrophages M1 polarization induced by sNPC coculture (Figure 4C,D). The RT‐qPCR analysis results revealed that iMSCs‐Exos markedly suppressed the expression level of pro‐inflammatory markers IL‐1β, IL‐6, and TNF‐α in M1 macrophages (Figure 4E). Besides, the flow cytometry analysis of M2 polarization marker CD206 showed that iMSCs‐Exos significantly increased the macrophages M2 polarization (Figure 4F,G). Subsequent RT‐qPCR analysis of M2 polarization markers IL‐10, CD163, and CD206 were consistent with the above findings (Figure 4H), which indicated that iMSCs‐Exos might promote the transition of sNPC‐induced M1 macrophages into anti‐inflammatory M2 macrophages. Moreover, we then investigated the effects of M1 macrophages with iMSCs‐Exos treatment on NP cell senescence. Compared to the M1 macrophages‐conditioned medium group, the NP cell proliferation level in the iMSCs‐Exos pretreated M1 macrophages‐conditioned medium group was significantly increased (Figure 4I,J). The SA‐β‐gal activity was also decreased in the iMSCs‐Exos pretreated M1‐CM group compared to the M1‐CM group (Figure 4K,L). The cell cycle analysis revealed that the cell proportion of G0/G1 phase in the iMSCs‐Exos pretreated M1 macrophages conditioned medium group was significantly decreased, and the cell proportion of S phase was markedly increased, compared to the M1 macrophages conditioned medium group (Figure S4, Supporting Information). The immunofluorescence analysis of cell senescence markers p16 and p21 demonstrated that the NP cell senescence level in the iMSCs‐Exos pretreated M1‐CM group was significantly decreased compared to the M1‐CM group (Figure 4M–P). Together, the above findings suggested that iMSCs‐Exos could promote re‐polarization of sNPC‐induced M1 macrophages to attenuate M1‐CM‐induced NP cells senescence.

### 2.5 iMSCs‐Exos Delivered miR‐100‐5p to Regulate Re‐Polarization of Macrophages

**Figure 5:**
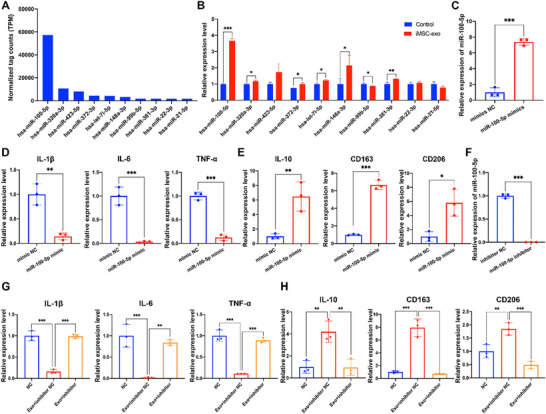
The miR‐100‐5p enriched in iMSCs‐Exos promoted the re‐polarization of M1 macrophages into M2 macrophages. A) The ten most abundant miRNAs enriched in iMSCs‐Exos were exhibited by the histogram based on miRNA sequencing. B) The macrophages were treated with or without iMSCs‐Exos, and the RNA expression levels of hsa‐miR‐100‐5p, hsa‐miR‐320a‐3p, hsa‐miR‐423‐5p, hsa‐miR‐372‐3p, hsa‐let‐7i‐5p, hsa‐miR‐148a‐3p, hsa‐miR‐99b‐5p, hsa‐miR‐381‐3p, hsa‐miR‐22‐3p, and hsa‐miR‐21‐5p in each group were detected by RT‐qPCR analysis. C) The macrophages were treated by mimic NC or miR‐100‐5p mimic, and the miR‐100‐5p RNA expression level was detected by RT‐qPCR analysis. D) The mRNA expression level of IL‐1β, IL‐6, and TNF‐α of the macrophages from each group was determined by RT‐qPCR analysis. E) The mRNA expression level of IL‐10, CD163, and CD206 of the macrophages from each group was determined by RT‐qPCR analysis. F) The macrophages were treated by inhibitor NC or miR‐100‐5p inhibitor, and the miR‐100‐5p RNA expression level was detected by RT‐qPCR analysis. G) The mRNA expression level of IL‐1β, IL‐6, and TNF‐α of the macrophages from each group was determined by RT‐qPCR analysis. H) The mRNA expression level of IL‐10, CD163, and CD206 of the macrophages from each group was determined by RT‐qPCR analysis.

To explore the mechanism of iMSCs‐Exos induced re‐polarization of macrophages, RNA‐seq was performed to analyze the gene expression within the exosomes, and relative expression levels of the top ten most abundant miRNAs in iMSCs‐Exos were shown in **Figure** **5**A. Subsequent RT‐qPCR analysis was performed to investigate the expression of these ten miRNAs in macrophages with or without iMSCs‐Exos treatment, and results showed that miR‐100‐5p was the most significantly up‐regulated miRNA with an approximate fourfold change (Figure 5B). Next, we transfected the macrophages with miR‐100‐5p mimics or inhibitors to over‐express or knock‐down miR‐100‐5p, to investigate the roles of miR‐100‐5p in regulating re‐polarization of macrophages. RT‐qPCR analysis confirmed that the miR‐100‐5p expression was significantly upregulated after miR‐100‐5p mimics treatment (Figure 5C). Moreover, miR‐100‐5p could significantly inhibit the mRNA expression of pro‐inflammatory markers IL‐1β, IL‐6, and TNF‐α in macrophages (Figure 5D). However, miR‐100‐5p obviously increased the expression levels of M2 polarization markers IL‐10, CD163, and CD206 in macrophages (Figure 5E). These data suggested that miR‐100‐5p could suppress macrophages M1 polarization and promote M2 polarization. Besides, the miR‐100‐5p expression was significantly downregulated after miR‐100‐5p inhibitor treatment compared to the NC group (Figure 5F). Further RT‐qPCR analysis of M1 polarization markers (IL‐1β, IL‐6, and TNF‐α) and M2 polarization markers (IL‐10, CD163, CD206) indicated that the effects of iMSCs‐Exos on macrophages re‐polarization were abolished by miR‐100‐5p inhibitor treatment (Figure 5G,H). Together, these findings revealed that iMSCs‐Exos could deliver miR‐100‐5p to inhibit macrophages M1 polarization and promote M2 polarization.

### 2.6 MiR‐100‐5p Regulated Polarization of Macrophages via mTORC1 Signaling and Glycolysis Metabolic Reprogramming

**Figure 6:**
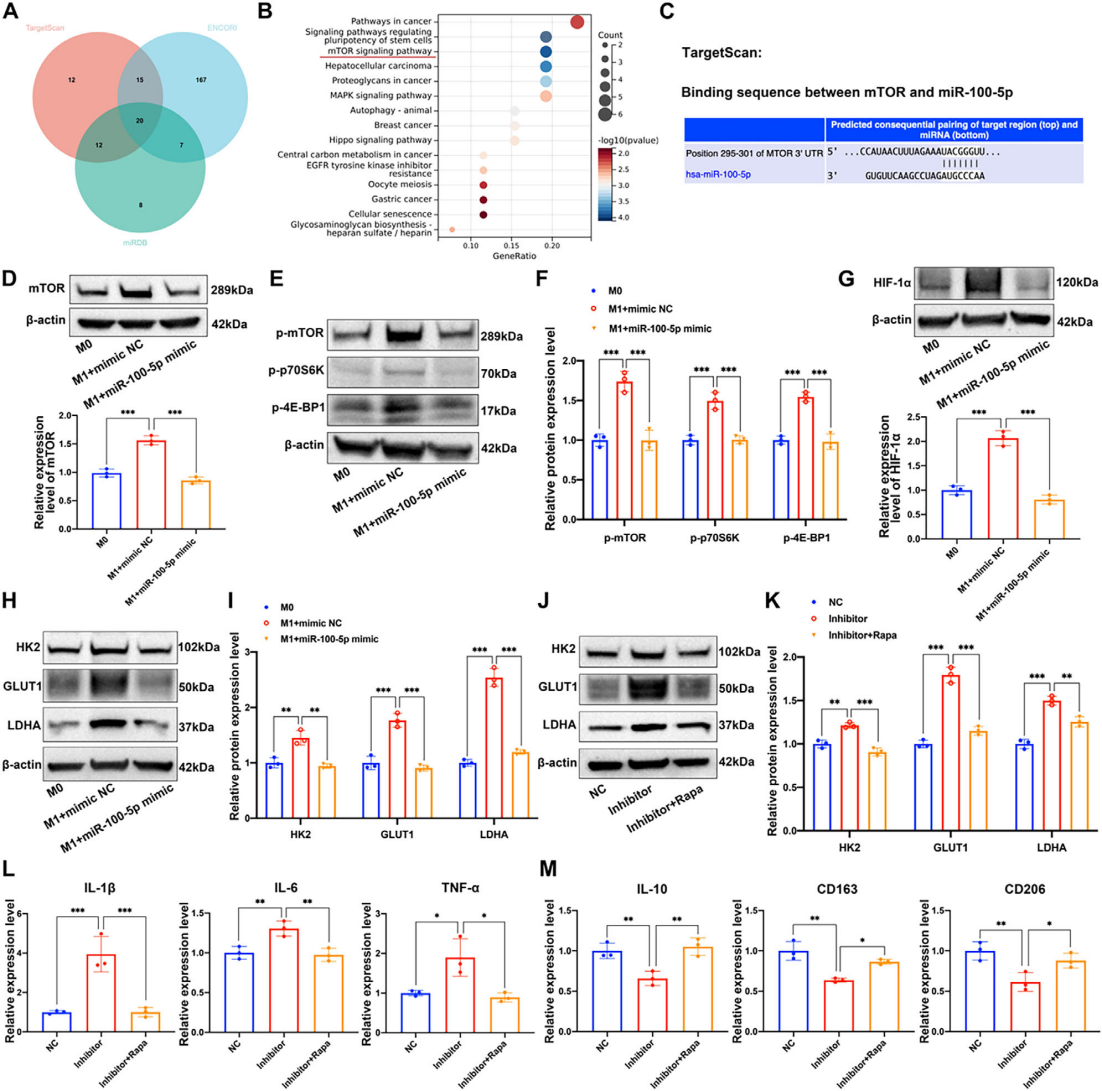
MiR‐100‐5p targeted mTOR and inhibited the mTORC1 signaling to regulate metabolic reprogramming in macrophages. A) Venn diagram showing the target genes of miR‐100‐5p as predicted by TargetScan, ENCORI, and miRDB. B) The top 15 most enriched KEGG pathways for these target genes of miR‐100‐5p. C) Schematic of the predicted binding site between the 3'‐UTR of mTOR mRNA and miR‐100‐5p based on the TargetScan online database. D) The M1 macrophages were pre‐treated with mimic NC or miR‐100‐5p mimic, and the protein expression level of mTOR in each group was assessed by western blot analysis. E,F) The protein expression levels of p‐mTOR, p‐p70S6K, and p‐4E‐BP1 in each group were assessed by western blot analysis. G) The protein expression level of HIF‐1α in each group were assessed by western blot analysis. H,I) The protein expression levels of HK2, GLUT‐1, and LDHA in each group were assessed by western blot analysis. J,K) The macrophages were pre‐treated with miR‐100‐5p inhibitor, or miR‐100‐5p inhibitor + Rapamycin, and the protein expression levels of HK2, GLUT‐1, and LDHA in each group were assessed by western blot analysis. L) The mRNA expression level of IL‐1β, IL‐6, and TNF‐α of the macrophages in each group was determined by RT‐qPCR analysis. M) The mRNA expression level of IL‐10, CD163, and CD206 of the macrophages in each group was determined by RT‐qPCR analysis.

MicroRNAs (miRNAs) are important epigenetic regulators that regulate gene expression post‐transcriptionally by guiding the RNA‐induced silence complex (RISC) to the 3'‐untranslated region (3'‐UTR) of target genes mRNA.^[^ ^25^ ^]^ Next, the potential target genes for miR‐100‐5p were predicted by TargetScan, ENCORI, and miRDB online database. Among the 20 target genes as shown in the Venn diagram (**Figure** **6**A), mTOR might be the most critical one which has played important roles in regulating macrophages M1 and M2 polarization.^[^ ^26^, ^27^ ^]^ Interestingly, the KEGG pathway analysis for these target genes of miR‐100‐5p also revealed that the mTOR signaling pathway was one of the most significantly enriched pathways (Figure 6B). The binding site between miR‐100‐5p and mTOR 3'‐UTR region was predicted by bioinformatics analysis based on the TargetScan database (Figure 6C), which has been already confirmed by the luciferase reporter gene assays previously.^[^ ^28^, ^29^ ^]^ Besides, we further performed RNA‐binding protein immunoprecipitation assays to validate the interaction of miR‐100‐5p and mTOR in macrophages, and RT‐qPCR analysis demonstrated that both miR‐100‐5p and mTOR were significantly enriched in AGO2‐conjugated beads compared to the IgG control group (Figure S5A, Supporting Information). These results indicated that miR‐100‐5p could unify with AGO2 protein to form the RISC to regulate mTOR expression. Moreover, RT‐qPCR analysis revealed that miR‐100‐5p mimics markedly inhibited the mRNA expression of mTOR in macrophages (Figure S5B, Supporting Information). Western blot assay demonstrated that miR‐100‐5p mimics could obviously decrease mTOR protein expression in macrophages compared to the NC group (Figure 6D). The mTOR signaling cascade consists of two multi‐subunit complexes called mTOR complex 1/2 (mTORC1/2) and previous studies have reported that mTORC1 is closely associated with macrophages M1 and M2 polarization.^[^ ^30^ ^]^ Then we further explored the effects of miR‐100‐5p on mTORC1 signaling in regulating macrophage polarization. The key marker of mTORC1 pathway activation p‐mTOR, and phosphorylation of the downstream substrates 4E‐BP1 and p70S6K were investigated. Results showed that miR‐100‐5p mimics significantly suppressed the protein expression of p‐mTOR, p‐p70s6k, and p‐4E‐BP1 compared to the NC group, indicating miR‐100‐5p could inhibit mTORC1‐p70S6K/4E‐BP1 signaling activation in macrophages (Figure 6E,F). Previous study has reported that mTORC1 could induce changes in glucose metabolism and lead to a shift from oxidative phosphorylation to glycolysis, which could promote M1 macrophage polarization.^[^ ^31^ ^]^ Then we investigated the effects of miR‐100‐5p on glycolysis in macrophages, and we found that miR‐100‐5p could markedly suppress the protein expression of hypoxia‐inducible factor 1‐alpha (HIF‐1α), hexokinase 2 (HK2), glucose transporter 1 (GLUT‐1), and lactate dehydrogenase A (LDHA) in macrophages (Figure 6G–I). Then miR‐100‐5p inhibitor and the mTORC1 inhibitor rapamycin (Rapa) was used to further validate these findings. We found that miR‐100‐5p inhibitor could facilitate the expression of HK2, GLUT‐1, and LDHA in macrophages, whereas these effects were abolished by inhibiting mTORC1 (Figure 6J,K). Subsequent RT‐qPCR analysis of M1 polarization markers (IL‐1β, IL‐6, and TNF‐α) and M2 polarization markers (IL‐10, CD163, CD206) demonstrated that miR‐100‐5p inhibitor promoted macrophages M1 polarization and inhibited M2 polarization. However, these effects of miR‐100‐5p inhibitor could be counteracted by rapamycin treatment (Figure 6L,M). These findings suggested that miR‐100‐5p inhibited macrophages M1 polarization and promoted M2 polarization via mTORC1 signaling and glycolysis metabolic reprogramming.

### 2.7 iMSCs‐Exos Delivered miR‐100‐5p to Regulate Macrophage Polarization and Mitigate IDD Progression In Vivo

**Figure 7:**
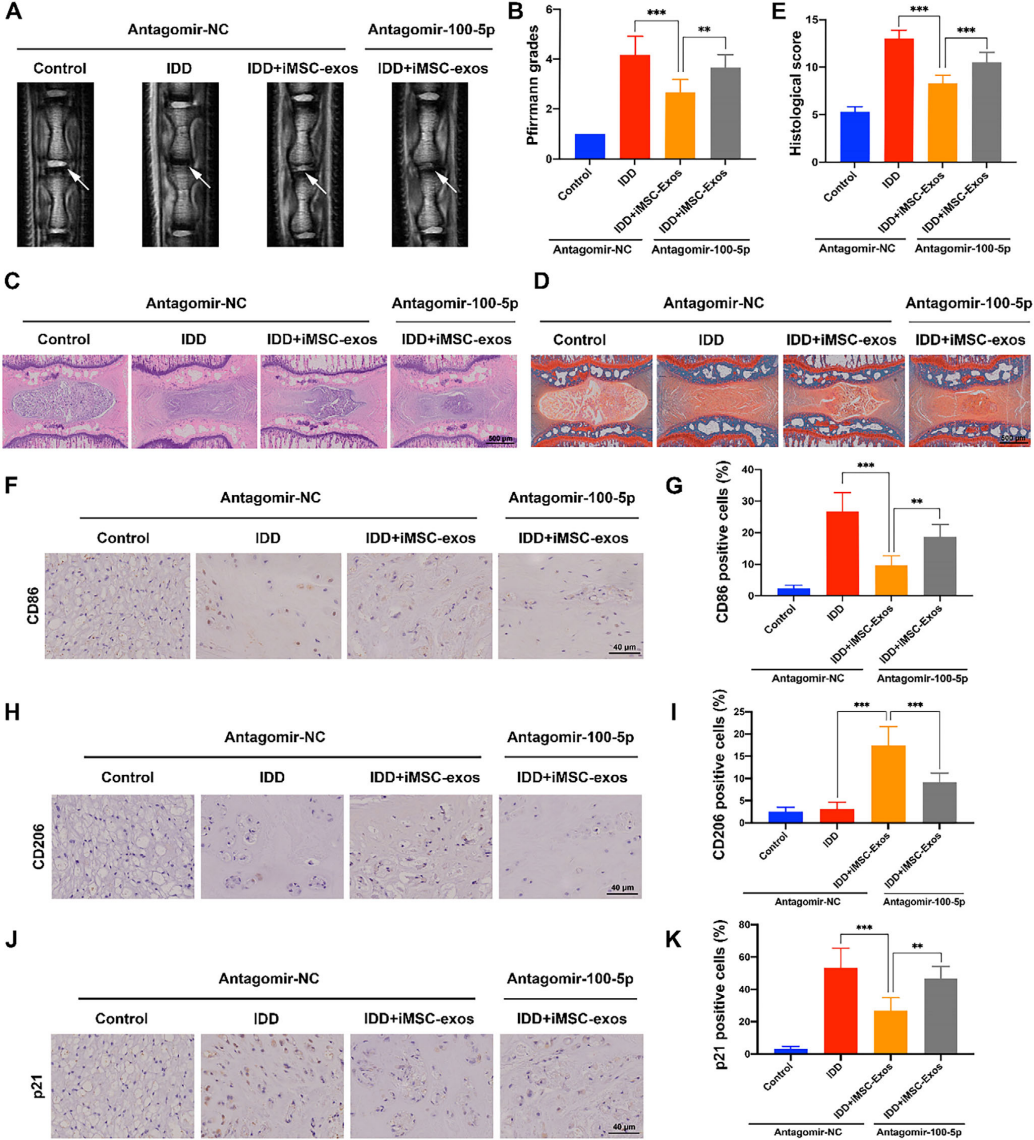
The iMSCs‐Exos regulated macrophages re‐polarization and inhibited NP cell senescence to mitigate IDD progression in vivo. A) The IVD from the rat tail from each group was evaluated by MRI at T2‐weighted signal (white arrows). B) Quantitation of the degree of IDD based on the Pfirrmann grade system. C) HE staining of the rat tail IVD from each treatment group. Scale bar: 500 µm. D) SO staining of the rat tail IVD from each treatment group. Scale bar: 500 µm. E) The histological score of the rat tail IVD from each group based on the histological grading scale. F,G) The protein expression level of CD86 in the rat tail IVD from each group was determined by immunohistochemical staining. Scale bar: 40 µm. H,I) The protein expression level of CD206 in the rat tail IVD from each group was determined by immunohistochemical staining. Scale bar: 40 µm. J,K) The protein expression level of p21 in the rat tail IVD from each group was detected by immunohistochemical staining. Scale bar: 40 µm.

The effects of iMSCs‐Exos on regulating macrophage polarization during IDD were further validated in the rat tail IDD model. The rat tail discs were injected with iMSC‐Exos combined with antagomir‐NC or antagomir‐100‐5p, and the degree of degeneration was evaluated by T2‐weighted MRI analysis. The MRI results suggested that the T2‐weighted signal value was higher in iMSC‐Exos treated group, and the Pfirrmann MRI grading score was lower in iMSC‐Exos treated group compared to the IDD group, indicating the protecting roles of iMSC‐Exos on the disc. However, the protecting effects of iMSC‐Exos in IDD were obviously abolished with the injection of antagomir‐100‐5p (**Figure** **7**A,B). By using HE and SO staining combined with histological score analysis to assess the morphological changes, we further found that the mitigating effects of iMSC‐Exos on the disc structure changes could be counteracted by miR‐100‐5p knockdown (Figure 7C–E). Moreover, immunohistochemical staining of CD86 in the disc demonstrated that iMSC‐Exos suppressed the M1 macrophages infiltration in IDD, which could be attenuated by antagomir‐100‐5p (Figure 7F,G). The immunohistochemical staining results of CD206 showed that iMSC‐Exos increased the cell proportion of M2 macrophages, which could be also counteracted by antagomir‐100‐5p additional treatment (Figure 7H,I). These results indicated that iMSCs‐Exos regulated macrophage polarization in a miR‐100‐5p‐dependent manner in vivo. Further immunohistochemical staining results of p21 confirmed that iMSC‐Exos decreased the p21‐positive cells proportion in the disc, and antagomir‐100‐5p obviously counteracted the effects of iMSC‐Exos on cell senescence in IVD (Figure 7J,K). All together, these findings suggested that iMSCs‐Exos promoted macrophages re‐polarization to mitigate cell senescence and IDD progression in vivo via miR‐100‐5p.

## 3 Discussion

**Figure 8:**
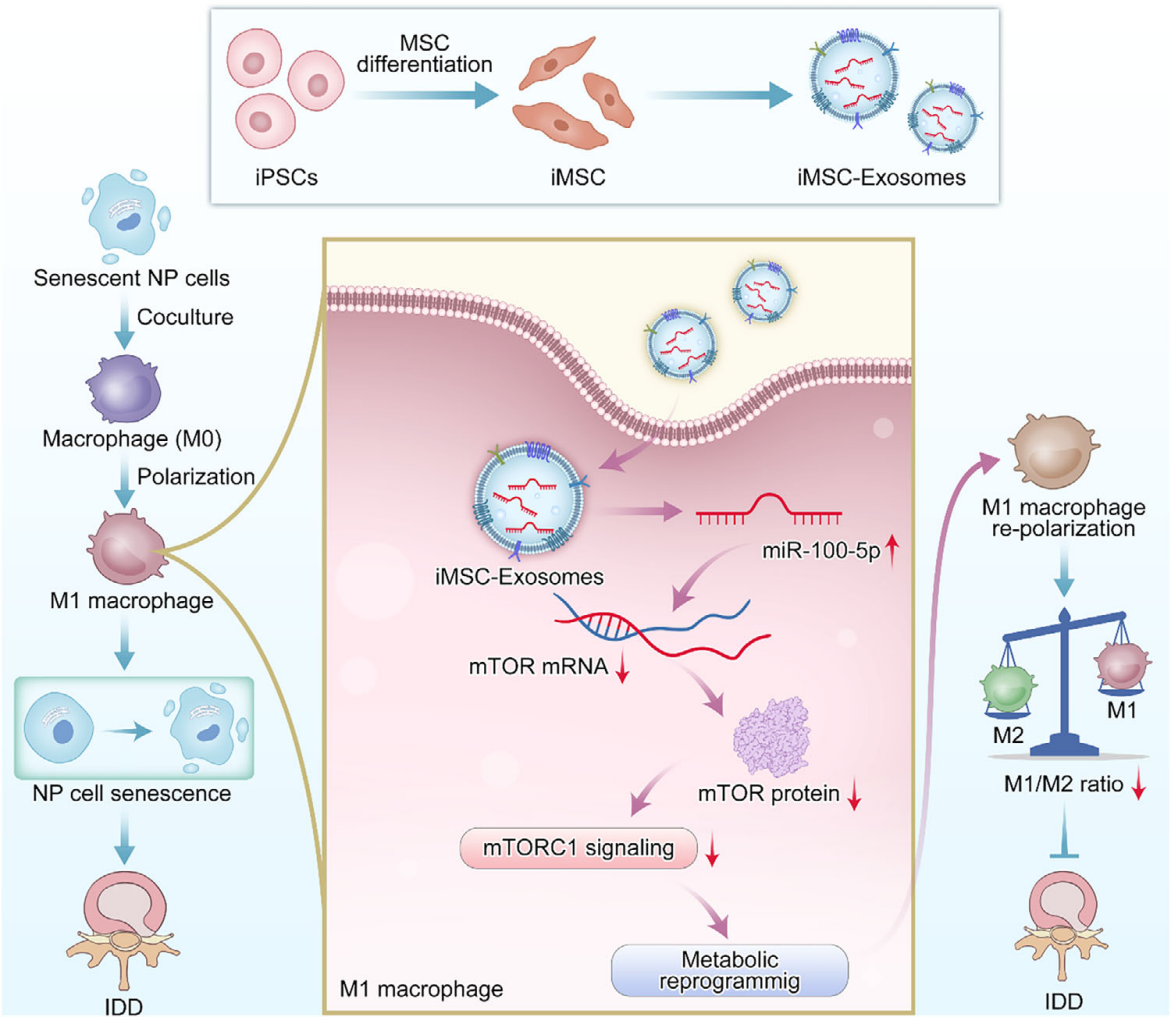
Schematic diagram of the interaction between the macrophage polarization and nucleus pulposus cell senescence in IDD progression, and the mechanism of iMSCs derived exosomes in modulating M1 macrophages re‐polarization to treat IDD. The iMSCs derived exosomes transport the enriched miR‐100‐5p, which target mTOR and inhibit the mTORC1 signaling to regulate metabolic reprogramming in macrophages.

Intervertebral disc degeneration is one of the most common degenerative spine diseases and is considered as a major contributor to lower back pain, which occurs in 80% of adults in their lifetime.^[^ ^1^, ^32^ ^]^ However, current clinical treatment for IDD‐related chronic back pain is limited to symptom relief, without effectively reversing the disc degeneration pathology, which might result in the recurrence of disc degeneration and dysfunction.^[^ ^32^, ^33^ ^]^ Therefore, it is of great importance to explore effective interventions to prevent IDD via targeting the underlying pathological mechanisms. The healthy IVD is an avascular and immune‐privileged organ away from macrophages.^[^ ^34^ ^]^ However, when neovascularization is established in the degenerated IVD, there exists a crosstalk between NP cells and macrophages.^[^ ^35^, ^36^ ^]^ Macrophages contribute to a chronic inflammatory micro‐environment in degenerative IVD, which has played important roles in the pathogenesis and progression of IDD. Previous studies have reported that macrophages could release various inflammatory mediators, including TNF‐α, IL‐1β, and IL‐6, which promoted the disc cell degeneration and deterioration.^[^ ^14^, ^15^ ^]^ M1‐type macrophages represent a crucial subset of pro‐inflammatory immune cells, and emerging evidence demonstrates a positive correlation between M1 macrophage polarization and IDD progression.^[^ ^11^, ^12^ ^]^ The interaction between macrophages and the NP cells is complex and might play vital roles in IDD progression. In this study, we validated that M1‐type macrophages infiltration and cell senescence were both elevated during IDD progression. We further investigated the interaction between macrophage polarization and NP cells senescence, and found that senescent NP cells significantly induced macrophages polarizing into the pro‐inflammatory M1 phenotype by using coculture experiments. Besides, we further uncovered that senescent nucleus pulposus cells‐induced M1 macrophages conditioned medium could promote normal NP cell senescence in return. In other words, our findings revealed that NP cell senescence could be transmitted and expanded by macrophage polarization for the first time. The schematic diagram of the interaction between macrophage polarization and NP cell senescence in IDD progression is shown in **Figure** **8**.

Considering the important roles of macrophage polarization on NP cell senescence in the disc degeneration process, we aimed to identify efficacious treatments that prevent IDD development by acting on macrophage polarization. Recently, the crucial roles of exosomes in immune regulation and tissue regeneration has been appreciated.^[^ ^37^, ^38^, ^39^ ^]^ Exosomes are a kind of natural extracellular vesicles with high biocompatibility and low toxicity, which play a critical role in intercellular communication. It has been demonstrated that MSCs‐derived exosomes could deliver many regenerative bioactive molecules to exert immune regulating functions and therapeutic effects in various human diseases.^[^ ^21^, ^40^, ^41^ ^]^ Interestingly, a previous study has reported that MSCs‐derived exosomes modulated macrophage polarization to mitigate acute lung injury by transferring miR‐27a‐3p,^[^ ^42^ ^]^ highlighting the crucial function of MSCs‐derived exosomes on macrophages related immune regulation. In this study, we employed a novel iPSC‐derived MSC population (iMSCs). And iMSCs‐derived exosomes overcome the critical limitations of conventional MSCs exosomes, offering higher consistency, scalability, and therapeutic potential. To the best of our knowledge, this study is the first to systematically investigate the function and mechanisms of iMSCs‐derived exosomes on macrophage polarization in IDD. We found that iMSCs‐Exos promoted the re‐polarization of M1 macrophages into anti‐inflammatory M2 phenotype, which could mitigate M1 macrophages‐induced NP cell senescence and treat IDD progression. In contrast to the previous study by Sun et al.^[^ ^43^ ^]^ which examined the direct effects of iMSCs‐derived extracellular vesicles (EVs) on NP cells in IDD, we identified and characterized a novel biological mechanism mediated by macrophages. We further analyzed the gene expression within the iMSCs‐Exos and unveiled the key roles of the most enriched miRNA miR‐100‐5p. Subsequently, the effects of miR‐100‐5p on macrophage polarization were confirmed by miR‐100‐5p mimics or inhibitor transfection. Furthermore, we found that miR‐100‐5p could target mTOR to inhibit its expression and thus to modulate macrophage polarization. The mTOR signaling cascade consists of two distinct multi‐subunit complexes called mTORC1 and mTORC2.^[^ ^44^, ^45^, ^46^ ^]^ In the present study, iMSCs‐Exos was found to suppress the mTORC1 signaling to regulate glycolysis in macrophages, at least partially by transferring miR‐100‐5p.

The mTOR is a protein kinase that controls many important cell activities to maintain the homeostasis, including cell proliferation, apoptosis, migration, metabolism, immune responses, etc.^[^ ^30^, ^47^, ^48^, ^49^, ^50^ ^]^ The mTOR orchestrates diverse cellular processes through its two functionally distinct complexes, mTORC1 and mTORC2. While mTORC2's regulatory roles remain incompletely characterized, mTORC1 exerts its biological effects in part through the phosphorylation and activation of two well‐defined downstream effectors, 4E‐BP1 and p70S6K.^[^ ^51^ ^]^ It has been reported that mTORC1 could induce changes in glucose metabolism and lead to a shift from oxidative phosphorylation to glycolysis.^[^ ^31^ ^]^ This metabolic reprogramming is known as Warburg effect and is characterized by an elevation of glucose uptake and the lactate production, even in the presence of oxygen.^[^ ^52^ ^]^ mTORC1 promotes the Warburg effect via regulating key gene expression and key enzymes activity in glycolysis, including hexokinase 2 (HK2) and lactate dehydrogenase A (LDHA).^[^ ^53^ ^]^ In the glycolysis process, glucosetransporter‐1 (GLUT1) is another important enzyme to facilitates glucose transport across the cell membrane. Besides, previous study has demonstrated that hypoxia‐inducible factor 1 alpha (HIF‐1α) could increase the expression of the glycolytic enzymes, and glucose transporters.^[^ ^54^ ^]^ Interestingly, it has been reported that mTORC1 activation was sufficient to increase the HIF1α expression levels under normoxic conditions.^[^ ^31^ ^]^ In all cellular activities, including cell proliferation and differentiation, there is a significant need for an adequate energy supply, which is closely associated with glycolysis metabolic reprogramming. It has been revealed that glycolysis metabolic reprogramming has significant impacts on macrophages M1 and M2 polarization.^[^ ^55^ ^]^ Particularly, M1‐type macrophages rely heavily on glycolysis.^[^ ^16^ ^]^ In this study, we found that the miR‐100‐5p enriched in iMSCs‐Exos could inhibit the mTORC1 signaling to suppress glycolysis, which further inhibited M1 polarization and promoted M2 polarization of macrophages. Furthermore, by using a rat IDD model, these effects of iMSCs‐Exos on macrophages metabolic reprogramming and re‐polarization to mitigate cell senescence and IDD progression were validated in vivo.

This study has several limitations. First, while we demonstrate that iMSCs‐Exos regulate macrophage polarization through miR‐100‐5p‐mediated mTORC1 signaling, the pleiotropic nature of miR‐100‐5p suggests it may simultaneously modulate multiple pathways. A comprehensive understanding of its regulatory network will require advanced RNA‐protein interaction analyses in future studies. Second, while iMSCs‐derived exosomes show therapeutic promise for IDD, natural exosomes may suffer from suboptimal targeting efficiency and tissue retention. Engineering approaches, such as modification with targeting peptides, could improve delivery efficacy.^[^ ^56^ ^]^ Finally, we utilized a well‐established needle puncture‐induced rat IDD model in this investigation. Importantly, rigorous long‐term safety evaluations in clinically relevant large‐animal models will be crucial before considering therapeutic translation.

## 4 Conclusion

In conclusion, our study uncovered the interaction between macrophage polarization and the NP cell senescence in IDD. Senescent NP cells significantly induced macrophages polarizing into the pro‐inflammatory M1 phenotype. Besides, we identified that senescent NP cells‐treated M1 macrophages conditioned medium could induce normal NP cell senescence in return. Moreover, our study highlighted the critical roles of iMSCs‐Exos in modulating macrophage polarization via glycolysis metabolic reprogramming to break the pathogenic senescence‐inflammation loop in IDD for the first time, suggesting a novel cell‐free therapeutic strategy for IDD treatment.

## 5 Experimental Section

### NP Tissue Samples Collection

The NP tissue samples were obtained from patients with idiopathic scoliosis and lumbar disc herniation undergoing spinal surgery. The degree of IDD was determined by the Pfirrmann MRI grading system, and the samples with a Pfirrmann grade of I or II were regarded as controls. Fresh tissue samples were used for primary NP cell isolation, and the others were immediately stored in liquid nitrogen for subsequent analysis. This study was approved by the Ethics Committee for Human Subjects of Peking University Third Hospital, and all methods were used in strict accordance with the approved guidelines.

### NP Cells Isolation and Culture

The fresh NP tissues were cut into ≈1 mm^3^ segments and rinsed twice with PBS. The tissue segments were digested using 0.25% Trypsin (Invitrogen, Carlsbad, CA, USA) for 0.5 h and then 0.2% type II collagenase (Solarbio, Beijing, China) for 3 h at 37 °C. The cells were washed using PBS and centrifuged at 1000 r for 5 min, and then were cultured in Dulbecco's Modified Eagle's Medium (HyClone, Thermo Scientific, USA) with 10% fetal bovine serum (Invitrogen, Carlsbad, CA, USA), and 1% penicillin/streptomycin (Sigma–Aldrich, St. Louis, MO, USA) at 37 °C in 5% CO2. The NP cells were digested by trypsinization when they reached 80–90% confluence, and then were passaged for cell expansion. The culture medium was renewed every three days. The NP cells from passage two were utilized in the subsequent experiments.

### Macrophages Cell Culture

The human monocyte/macrophage THP‐1 cell line was cultured in six‐well plates at a density of 6 × 10^5^/mL. Then the cells were differentiated with 50 ng mL^−1^ of phorbol 12‐myristate 13‐acetate (PMA) (Sigma–Aldrich, USA) for 72 h to obtain M0 macrophages. Macrophages were cultured in RPMI‐1640 medium before subsequent treatments. For the conditioned medium (CM) collection, the culture medium was changed to serum‐free RPMI 1640 medium for 24 h after co‐culture with normal NP cells (nNPC) or senescent NP cells (sNPC) for 24 h. The CM of macrophages, including nNPC‐M0‐CM and nNPC‐M1‐CM were stored at −80 °C for the following experiments.

### Flow Cytometry Analysis

Flow cytometry analysis was performed to evaluate the polarization of macrophages with corresponding treatments. CD86 was used as the marker of macrophages M1 phenotype, and CD206 was used as the marker of macrophages M2 phenotype. In brief, macrophages were washed and suspended in cell staining buffer, and then were Fc‐blocked with Human TruStain FcX (BioLegend) for 10 min, according to the manufacturer's instructions. Then the cells were stained with APC‐labeled anti‐CD86 and PE‐labeled anti‐CD206 antibodies for 20 min away from light. After washing and re‐suspending with cell staining buffer, the cells were examined by flow cytometry.

### The iMSCs Culture

The iMSCs culture was performed as described previously.^[^ ^57^ ^]^ Briefly, the human induced pluripotent stem cells (cell line DYR0100) were grown on ESC‐Qualified Matrigel (BD Biosciences, NJ, USA) with ncTarget hPSC medium (Shownin Biotech, Hefei, China) in six‐well plates (NEST, Wuxi, China). When the iPSCs grown to 80% confluence, the culture medium was changed into MSC differentiation medium (Nuwacell, Hefei, China). After differentiation culture for two weeks, the cells were digested with 0.25% trypsin‐EDTA. Then the cells were seeded at a density of 5 × 10⁴ /mL in MSC culture medium using 0.1% gelatin coated culture flasks. The iMSCs from passage 3 displayed a typical fibroblast‐like morphology generally, and then were subjected to surface marker analysis by flow cytometry analysis.

### Isolation and Characterization of iMSC‐Exos

The exosomes were collected from cell culture supernatant as described previously.^[^ ^57^ ^]^ In brief, the iMSCs were grown for 48 h in serum‐free MSC medium. Then the conditioned medium was collected and centrifuged twice for non‐adherent cell extraction at 300×g for 10 min and again for cell and debris extraction at 1000×g for 15 min. Larger vesicles were extracted from the supernatant by centrifugation at 10 000×g for 1 h. The residual supernatant was centrifuged at 100,000×g for 70 min. The exosome pellet obtained was re‐suspended in PBS and stored at −80 °C. The BCA protein assay kit (PC0020, Solarbio, Beijing, China) was applied to determine the protein concentration of exosomes according to the manufacturer's instructions. The exosome markers (CD81, HSP70, TSG101, and Calnexin) were examined by Western blot. The morphology of the exosomes was observed using a transmission electron microscope (TEM, JEM‐1200EX, JEOL Ltd., Tokyo, Japan) at an acceleration voltage of 100 keV. The size distribution and concentration of the particles were assessed by nanoparticle tracking analysis (NTA) using ZetaView (Particle Metrix, Meerbusch, Germany).

### Cytotoxicity Analysis In Vitro

The cell viability of macrophages was determined by using a cell counting kit‐ (CCK‐) 8 assay (Dojindo, Japan). In brief, the macrophages were incubated with iMSC‐derived exosomes at different concentrations for 24 h, and then 20 µL of CCK‐8 solution was added to each well with 200 µL culture medium. The cells were then incubated at 37 °C for 2 h, and then the absorbance signal at 450 nm was detected using the SpectraMax iD3 spectrophotometer (Molecular Devices).

### Uptake of iMSC‐Exos by Macrophages

The purified iMSC‐derived exosomes were incubated with PKH67 (D0031, Solarbio, Beijing, China) for 5 min at room temperature. Next, the iMSC‐Exos were washed with PBS and centrifuged, and then were suspended in basal medium and incubated with macrophages for 12 h at 37 °C. The nuclei were stained with DAPI (K2401, APExBIO, Houston, USA) for 5 min. A fluorescence microscope (Nikon, Tokyo, Japan) was used for subsequent imaging.

### RNA Extraction and RT‐qPCR Analysis

Total RNA was isolated by a SteadyPure Universal RNA Extraction Kit (AG21017, Accurate Biology, China). The cytoplasmic and nuclear RNA for subcellular fractionation was isolated by using the PARIS Kit (AM1921, Life Technologies, USA). The RNA purity and concentration were assessed using a DHS NanoPro 2020 spectrophotometer. RNA was reverse‐transcribed to cDNA by using an Evo M‐MLV Mix Kit with gDNA Clean for qPCR (AG11728, Accurate Biology, China). The qPCR assay was conducted using SYBR Green Premix Pro Taq HS qPCR Kit (ROX Plus) (AG11718, Accurate Biology, China) on QuantStudio 3 Real‐Time PCR System (Applied Biosystems, USA). The qPCR conditions were set as follows: 95°C for 30 s, 40 cycles of 95 °C for 5 s, and 60 °C for 30 s. Then a melt curve stage was added after the PCR amplification stage, which was set as 95 °C for 15 s, 60 °C for 1 min, and 95 °C for 15 s. GAPDH was used as the internal control and relative expression levels of genes were calculated by using the comparative threshold cycle (Ct) method using the formula 2^−ΔΔCT^. The primers were synthesized by Sangon Biotech (Shanghai, China). Primers sequence used in this study were listed in Table S1 (Supporting Information).

### Transfection Vector Construction and Cell Transfection

The hsa‐miR‐100‐5p mimic, hsa‐miR‐100‐5p inhibitor, and antagomir‐100‐5p were designed and synthesized by Sangon Biotech (Shanghai, China). For cell transfection, the cells were seeded in 6‐well plates until 80% confluence, and then transfections were conducted by using Lipofectamine 3000 Transfection Reagent (Invitrogen, USA), according to the manufactures' instructions. The transfection efficiency was verified at 24 h after transfection, followed by cell treatments. Experiments were performed three times independently.

### Western Blot Analysis

After removing the medium and washing with PBS, the total protein of cells was extracted using a Protein Extraction Kit (BC3710, Solarbio, Beijing, China) according to the protocol described. The protein concentration of cell lysate was evaluated by the BCA protein assay kit (PC0020, Solarbio, Beijing, China). Then SDS‐PAGE gels were applied to separate protein samples, which were subsequently transferred to PVDF membranes (Millipore, USA). The membranes were blocked for 1 h and then incubated with specific primary antibodies (1:500‐1:1000) overnight at 4 °C. After washing with TBST buffer for three times, membranes were incubated with a secondary antibody (1:2000, Abcam) at 25 °C for 1 h. Primary antibodies against the following proteins were used in this study: p21 (10355‐1‐AP, Proteintech, USA), p16 (A0262, ABclonal, China), CD81 (ab109201, Abcam, USA), HSP70 (ab181606, Abcam, USA), Calnexin (ab133615, Abcam, USA), TSG101 (ab125011, Abcam, USA), mTOR (2983T, CST, USA), p‐mTOR (5536T, CST, USA), p‐p70s6k (9234T, CST, USA), p‐4E‐BP1 (2855T, CST, USA), HIF‐1α (36169T, CST, USA), GLUT‐1 (73015S, CST, USA), HK2 (2867T, CST, USA), LDHA (3582T, CST, USA), β‐actin (AF5003, Beyotime, China). The protein expression was observed with the iBright CL1000 imaging system (Invitrogen, USA).

### EdU Staining Analysis

The EdU staining was analyzed by using the E‐Click EdU Cell Proliferation Imaging Assay Kit (E‐CK‐A377, Elabscience, China). The NP cells were cultured with EdU before fixation and permeabilization, and the following labeling and nuclear staining procedures were performed according to the manufacturer’ instructions. A fluorescence microscope (Nikon, Tokyo, Japan) was used for subsequent imaging.

### SA‐β‐gal Activity Analysis

The senescence‐associated β‐galactosidase (SA‐β‐gal) activity of NP cells was determined by the Senescence β‐Galactosidase Staining Kit (C0602, Beyotime, China) based on the manufacturer's protocol. After fixation and PBS washing, cells were treated by the staining working solution overnight at 37 °C. SA‐β‐gal activity was expressed as the percentage of SA‐β‐gal staining‐positive cells to total cells.

### Cell Cycle Analysis

The NP cells from each experimental group were harvested from 6‐well plates and processed for cell cycle analysis by using a Cell Cycle Staining Kit (MultiSciences, China). Briefly, the NP cells were washed with PBS, resuspended at a density of 1×10^6^ cells mL^−1^, and fixed overnight in 70% ice‐cold ethanol at 4 °C. After fixation, cells were washed with PBS to remove ethanol and incubated with the staining solution away from light at room temperature for 60 min. Finally, DNA content was analyzed by flow cytometry to determine the distribution of cells in G0/G1, S, and G2/M phases.

### Immunofluorescent Analysis

The NP cells were first fixed with 4% paraformaldehyde, and then were treated with the Immunol Staining Blocking Buffer (P0102, Beyotime, China) at room temperature for 1 h. Next, the slides were incubated with the corresponding primary antibodies (p16, 1:200, A0262, ABclonal; p21, 1:200, 10355‐1‐AP, Proteintech) diluted in primary antibody dilution buffer (P0262, Beyotime, China) overnight at 4 °C. After washing with PBS, the slides were treated with a secondary antibody labeled with Alexa Fluor 594 (1:400, 33112ES60, Yeasen, China) at room temperature for 2 h. Nuclei were stained with DAPI (K2401, APExBIO, Houston, USA). A fluorescence microscope (Nikon, Tokyo, Japan) was used for subsequent imaging.

### Animal Experiments

The animal experimental protocol was approved by the Institutional Animal Care and Use Committee of Peking University Health Science Center (DLASBD0216). A rat tail puncture‐induced IDD model was established as described previously.^[^ ^22^ ^]^ Briefly, 6‐week‐old Sprague‐Dawley rats were anaesthetized using 3% (w/v) pentobarbital. The skin was disinfected, and the rats Co 8/9 coccygeal disc was punctured by a 20‐gauge needle through the tail skin from the dorsal side to induce disc degeneration. The rats were randomly divided into corresponding groups and administrated with or without 100 µg mL^−1^ iMSC‐Exos, or combined with antagomir‐nc or antagomir‐100‐5p, by using a 33‐gauge needle to minimize the effect of repeated puncture. The injection volume was set as 2 µL and the corresponding procedure was repeated every week for 4 weeks.

### Magnetic Resonance Imaging (MRI) Analysis

Magnetic Resonance Imaging (MRI) analysis was performed on the rat model coccygeal disc by using a 3.0 T MRI system (Siemens, Germany) to evaluate the disc structure. The severity of IDD was determined by median coronal T2‐weighted images of the discs based on the modified Pfirrmann grading system.^[^ ^24^ ^]^

### Histological Analysis and Immunohistochemistry Staining

The rat IVD samples were collected and washed by PBS, and then were fixed in formaldehyde. After slowly decalcifying with 10% EDTA for 3 weeks, the tissues were dehydrated and embedded in paraffin. Then the paraffin block samples were sectioned in the sagittal plane at a thickness of 5 µm. Sections were then stained with hematoxylin‐eosin (HE) or safranin O‐fast green (SO) solution. For immunohistochemistry staining, the procedures were performed as described previously.^[^ ^58^ ^]^ In brief, after deparaffinization and rehydration, the sections were incubated with primary antibodies against mTOR (1:200), CD86 (1:500), CD206 (1:500), and p21 (1:200) at 4 °C overnight. Then the samples were incubated with HRP‐conjugated secondary antibodies and counterstained with hematoxylin.

### Bioinformatics Analysis

The target genes of hsa‐miR‐100‐5p were predicted by TargetScan: https://www.targetscan.org/vert_72/, ENCORI/starBase (Encyclopedia of RNA Interactomes): https://rnasysu.com/encori/, and miRDB: https://mirdb.org/. The KEGG analysis was conducted by the R package “clusterProfiler” based on the latest KEGG pathway genes annotations, which were obtained from the KEGG rest API (https://www.kegg.jp/kegg/rest/keggapi.html). The p‐value < 0.05 was considered statistically significant. The enriched items were plotted by using the R package “ggplot2”.

### Statistical Analysis

The data were analyzed by GraphPad Prism software version 9.0. All data were presented as means ± standard deviation (SD) of at least three independent experiments unless otherwise specified. Statistical difference analysis was conducted by unpaired Student's *t*‐test between two groups, or one‐way ANOVA test followed by Tukey post hoc test between multiple groups. The P value less than 0.05 was considered as statistically significant.

## Conflict of Interest

The authors declare no conflict of interest.

## Author Contributions

Q.X. and J.Z. contributed equally to this work. W.L. designed the study. Q.X., J.Z. and S.T. performed the experiments and collected the data. J.Z., S.T., Y.Z., Z.W., J.L., L.C., and L.W. analyzed the data. Q.X. drafted the manuscript. S.J., Z.S. and W.L. revised the manuscript. All authors approved the final version of the manuscript.

## Supporting information



Supporting Information

## Data Availability

The data that support the findings of this study are available from the corresponding author upon reasonable request.
