# Supplementary material for: Human iPSCs Derived MSCs‐Secreted Exosomes Modulate Senescent Nucleus Pulposus Cells Induced Macrophage Polarization via Metabolic Reprogramming to Mitigate Intervertebral Disc Degeneration
Source: Adv Sci (Weinh). 2025 Jul 6;12(36):e04347. doi: 10.1002/advs.202504347 (PMC12463037; doi:10.1002/advs.202504347)
Supplement: Supplementary file 1 — Supporting Information [file ADVS-12-e04347-s001.pdf]

ADVANCED SCIENCE
Open Access

Supporting Information

for *Adv. Sci.*, DOI 10.1002/advs.202504347

Human iPSCs Derived MSCs-Secreted Exosomes Modulate Senescent Nucleus Pulposus Cells Induced Macrophage Polarization via Metabolic Reprogramming to Mitigate Intervertebral Disc Degeneration

*Qian Xiang, Jiawen Zhan, Shuo Tian, Yongzhao Zhao, Zhenquan Wu, Jialiang Lin, Longting Chen, Longjie Wang, Shuai Jiang, Zhuoran Sun and Weishi Li**

Supporting Information

Human iPSCs derived MSCs-secreted exosomes modulate senescent nucleus pulposus cells induced macrophage polarization via metabolic reprogramming to mitigate intervertebral disc degeneration

*Qian Xiang, Jiawen Zhan, Shuo Tian, Yongzhao Zhao, Zhenquan Wu, Jialiang Lin, Longting Chen, Longjie Wang, Shuai Jiang, Zhuoran Sun, Weishi Li**

This PDF file includes:

Figures S1 to S5

Table S1

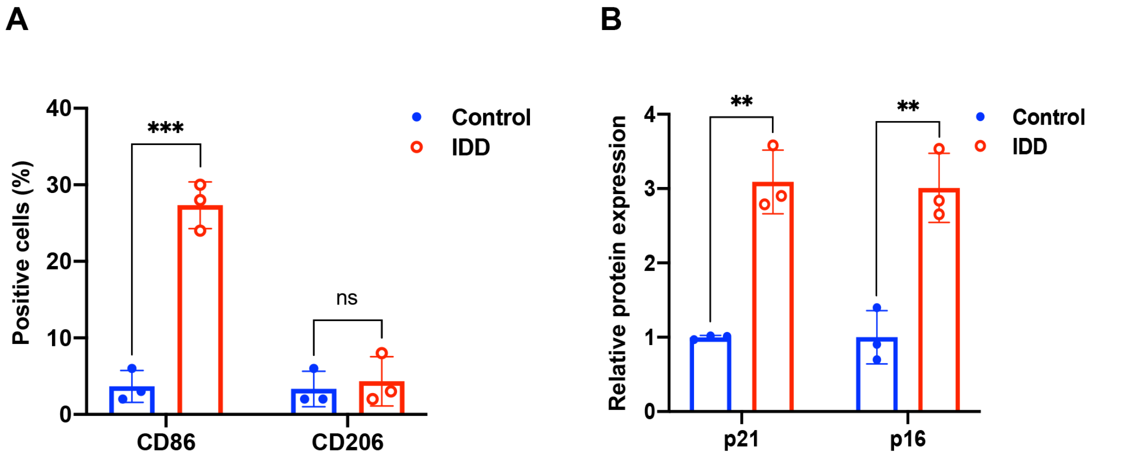

Figure S1. The M1 macrophages proportion and cell senescence level is up-regulated in IDD, related to Figure 1. (A) The quantitative analysis of CD86 and CD206 determined by immunohistochemical staining of NP tissues in control and IDD groups. (B) The quantitative analysis of p21 and p16 protein expression levels determined by western blot in control and IDD groups. ns (not significant), $^{**}p < 0.01$, $^{***}p < 0.001$, n = 3.

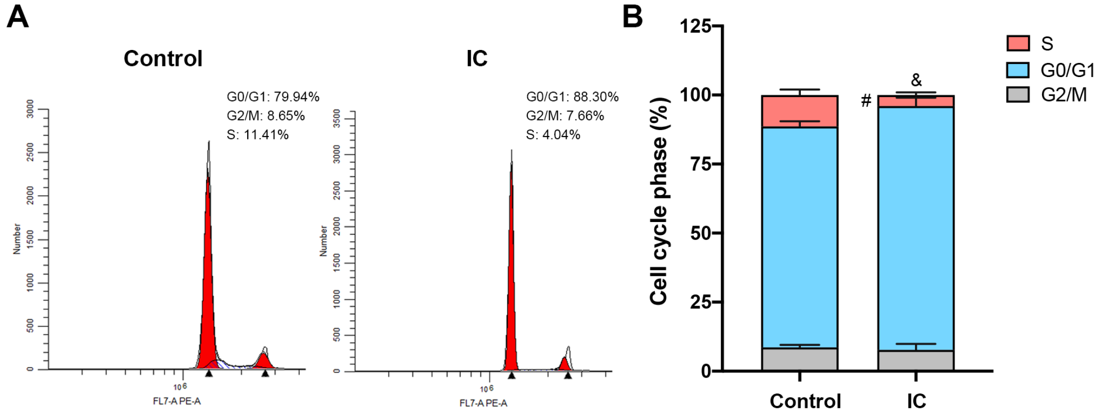

Figure S2. Inflammatory cytokines treatment significantly induced NP cells senescence in vitro, related to Figure 1. (A) The cell cycle distribution of NP cells in control or inflammatory cytokines (IC) treatment group was determined by flow cytometry. (B) The cell proportion of the G0/G1, S, and G2/M cell cycle phase of NP cells in each group. $^{\&}p < 0.05$ indicates statistical significance for comparing cell proportion in S phase, $^{\#}p < 0.05$ indicates statistical significance for comparing cell proportion in G0/G1 phase, n=3.

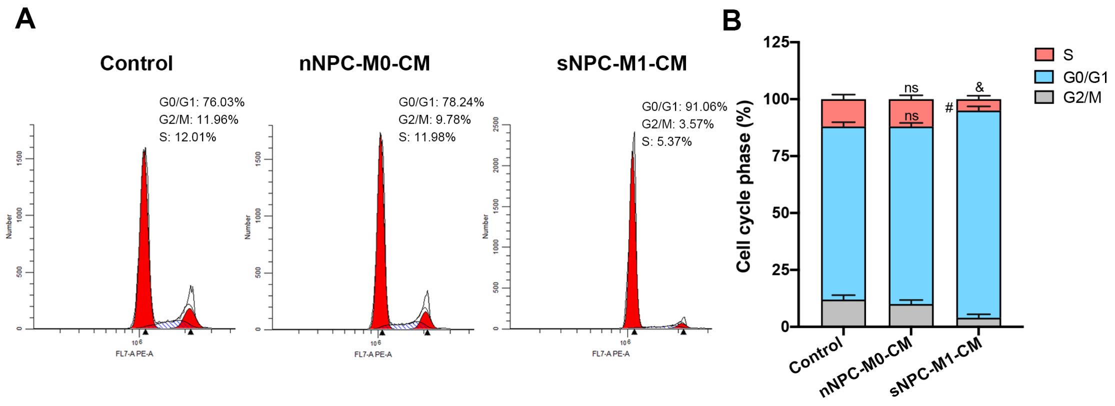

Figure S3. The M1 macrophages conditioned medium significantly promoted NP cells senescence, related to Figure 2. (A) The cell cycle distribution of NP cells in each group was determined by flow cytometry. (B) The cell proportion of the G0/G1, S, and G2/M cell cycle phase of NP cells in each group. ns, not significant; $^{\&}p < 0.05$ indicates statistical significance for comparing cell proportion in S phase, $^{\#}p < 0.05$ indicates statistical significance for comparing cell proportion in G0/G1 phase, n=3.

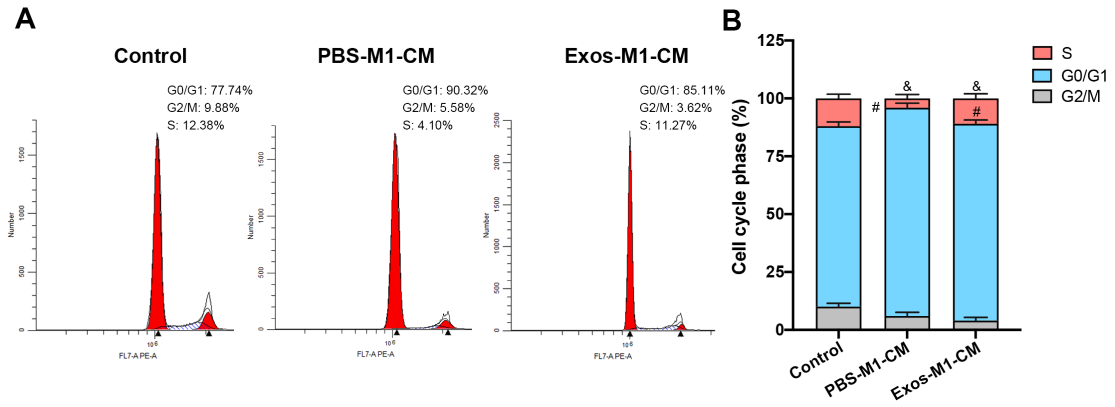

Figure S4. Treatment wtih iMSCs-Exos attenuated M1 macrophages conditioned medium induced NP cells senescence, related to Figure 4. (A) The cell cycle distribution of NP cells in each group was determined by flow cytometry. (B) The cell proportion of the G0/G1, S, and G2/M cell cycle phase of NP cells in each group. ns, not significant; $^{\&}p < 0.05$ indicates statistical significance for comparing cell proportion in S phase, $^{\#}p < 0.05$ indicates statistical significance for comparing cell proportion in G0/G1 phase, n=3.

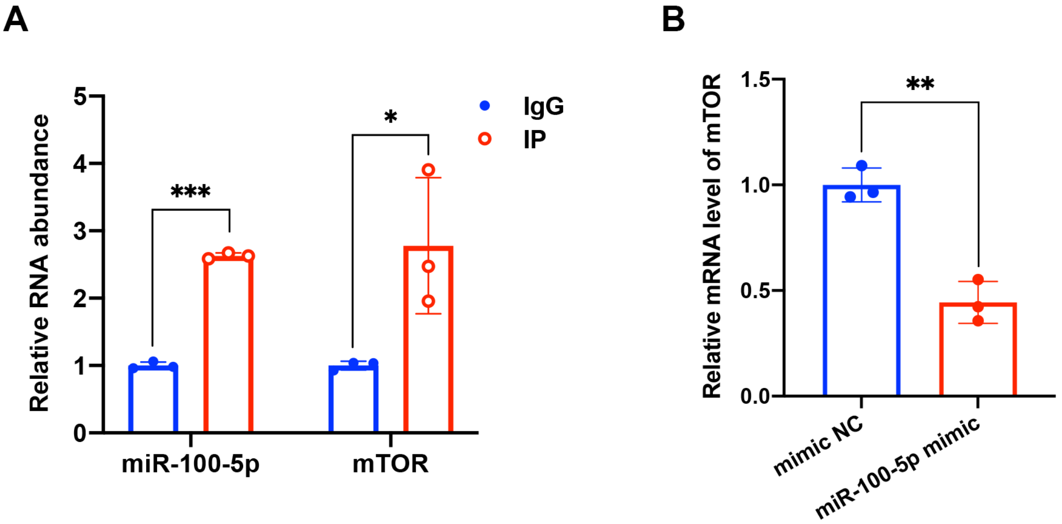

Figure S5. MiR-100-5p targeted mTOR to regulate its expression, related to Figure 6. (A) The miR-100-5p and mTOR mRNA abundance level of the RNA-binding protein immunoprecipitation assays conducted using anti-AGO2 (IP group) or anti-IgG (IgG group) antibody were determined by RT-qPCR analysis. (B) The effect of miR-100-5p on the mRNA expression of mTOR was assessed by RT-qPCR analysis. *$p < 0.05$, **$p < 0.01$, ***$p < 0.001$, $n = 3$.

Table S1. Primers used in this study

**Primers for RT-qPCR**		
IL-1β	Forward	ATGATGGCTTATTACAGTGGCAA
IL-1β	Reverse	GTCGGAGATTCGTAGCTGGA
IL-6	Forward	ACTCACCTCTTCAGAACGAATTG
IL-6	Reverse	CCATCTTTGGAAGGTTCAGGTTG
TNF-α	Forward	CCTCTCTCTAATCAGCCCTCTG
TNF-α	Reverse	GAGGACCTGGGAGTAGATGAG
IL-10	Forward	GACTTTAAGGGTTACCTGGGTTG
IL-10	Reverse	TCACATGCGCCTTGATGTCTG
CD163	Forward	AAAGAATCCCGCATTTGGCAGTG
CD163	Reverse	CAGATAACTCCCGCATCCTCCTTG

CD206	Forward	ACCTCACAAGTATCCACACCATCG
CD206	Reverse	GGGTCCCATCACTCCACTCAAAG
mTOR	Forward	CTGGGACTCAAATGTGTGCAGTTC
mTOR	Reverse	GAACAATAGGGTGAATGATCCGGG
Hsa-miR-100-5p	Forward	GCAACCCGTAGATCCGAACTTGTG
Hsa-miR-320a-3p	Forward	TAAAAGCTGGGTTGAGAGGGCGA
Hsa-miR-423-5p	Forward	TTATTGAGGGGCAGAGAGCGAGA
Hsa-miR-372-3p	Forward	AAAGTGCTGCGACATTTGAGCGT
Hsa-let-7i-5p	Forward	CGCTGAGGTAGTAGTTTGTGCTGTT
Hsa-miR-148a-3p	Forward	CCGTCAGTGCACTACAGAACTTTGT
Hsa-miR-99b-5p	Forward	CATTACACCCGTAGAACCGACCTTG
Hsa-miR-381-3p	Forward	CCCTATACAAGGGCAAGCTCTCTGT
Hsa-miR-22-3p	Forward	CGAAGCTGCCAGTTGAAGAACTGT
Hsa-miR-21-5p	Forward	CCCGGTAGCTTATCAGACTGATGTTGA
GADPH	Forward	ATGGGGAAGGTGAAGGTCG
GADPH	Reverse	GGGGTCATTGATGGCAACAATA
U6	Forward	CTCGCTTCGGCAGCACA
U6	Reverse	AACGCTTCACGAATTTGCGT
